# Nuclear Exportin 1 (XPO1) Binds to the Nuclear Localization/Export Signal of the Turnip Mosaic Virus NIb to Promote Viral Infection

**DOI:** 10.3389/fmicb.2021.780724

**Published:** 2022-01-04

**Authors:** Mingzhen Zhang, Pan Gong, Linhao Ge, Yinzi Li, Zhaoyang Chang, Rui Qiao, Xueping Zhou, Aiming Wang, Fangfang Li

**Affiliations:** ^1^State Key Laboratory for Biology of Plant Diseases and Insect Pests, Institute of Plant Protection, Chinese Academy of Agricultural Sciences, Beijing, China; ^2^London Research and Development Centre, Agriculture and Agri-Food Canada, London, ON, Canada; ^3^Department of Biology, Western University, London, ON, Canada; ^4^State Key Laboratory of Rice Biology, Institute of Biotechnology, Zhejiang University, Hangzhou, China

**Keywords:** nuclear localization signal (NLS), nuclear export signal (NES), NIb, turnip mosaic virus (TuMV), exportin 1 (XPO1)

## Abstract

The nuclear localization signal (NLS) and nuclear export signal (NES) are key signatures of proteins for controlling nuclear import and export. The NIb protein of turnip mosaic virus (TuMV) is an RNA-dependent RNA polymerase (RdRP) that is absolutely required for viral genome replication. Previous studies have shown that NIb is a nucleocytoplasmic shuttling protein and contains four putative NES and four putative NLS motifs. Here, we analyzed the function of these NESs and NLSs, and identified two functional NESs and one functional NLS. Mutation of the identified functional NESs or NLS inhibited viral RNA accumulation and systemic infection. Exportin 1 (XPO1) is a nuclear export receptor that binds directly to cargo proteins harboring a leucine-rich NES and translocates them to the cytoplasm. We found that XPO1 contains two NIb-binding domains, which recognize the NLS and NES of NIb, respectively, to mediate the nucleocytoplasmic transport of NIb and promote viral infection. Taken together, these data suggest that the nucleocytoplasmic transport of NIb is modulated by XPO1 through its interactions with the functional NLS and NES of NIb to promote viral infection.

## Introduction

Nuclear import and export that govern substrate exchange between the nucleus and the cytoplasm are crucial processes in any eukaryotic cell. The nucleocytoplasmic transport depends on a network of proteins that shuttle between the nucleus and cytoplasm, allowing substrate exchange through the nuclear pore complex (NPC). The NPC is a large, multi-subunit structure of ∼110–125 MDa in metazoans and consists of 8–64 copies of about 34 different nuclear pore proteins (Knockenhauer and Thomas, 2016; Lin et al., 2016; Ungricht and Ulrike, 2017; Shen et al., 2021). The plant NPC has similar structure and components to metazoans or yeast (Tamura et al., 2010; Tamura and Hara-Nishimura, 2013). The NPC permits passive diffusion of ions and small molecules up to 9 nm in diameter or up to 70 kDa for globular proteins to gain access to the nucleus (Görlich and Kutay, 1999). However, this diffusion is reasonably fast only for proteins of up to 20–30 kDa, and molecules and multimolecular complexes larger than 60 kDa must rely exclusively on energy-driven (active) mechanisms that are facilitated by a nuclear localization signal (NLS) or a nuclear export signal (NES) (Görlich and Kutay, 1999; Weis, 2003; Merkle, 2011).

The “classical” active nuclear import pathway begins with a pair of nuclear import receptors importin α and importin β and is ferried across the pore (Meier, 2005; Shen et al., 2021). In the cytoplasm, the importin α/β heterodimer using the NLS-binding region of importin α binds to a protein containing NLS. This trimeric complex is docked to the cytoplasmic face of the NPC and targeted to its core through the affinity of importin β for the NPC components (Nigg, 1997). Translocation into the nucleus requires recruitment of the GTP-bound small GTPase Ran (Ran-GTP) to importin β, which causes the disassembly of the import complex and releases the cargo. Furthermore, the complex of importin β and Ran-GTP is transported back to the cytoplasm, whereas importin α is recycled (Shen et al., 2021). In addition to the members of the importin (Imp) superfamily, an ever-expanding repertoire of Imp-independent nuclear import pathways/mechanisms such as the calcium-binding protein calmodulin or through direct binding to the components of the NPC has been found (Wagstaff and Jans, 2009). For nuclear export, nuclear export receptors such as exportin 1 (XPO1) [also known as chromosome maintenance region 1 (CRM1)] have been identified in different eukaryotic organisms to export substrates from the nucleus to the cytoplasm by targeting the cargo’s NES. During the export process, Ran-GTP in the nucleus stimulates binding of XPO1 to the export substrate. After translocation through the NPC, the XPO1/Ran-GTP/cargo protein complex is disassembled following dissociation of Ran-GTP that is hydrolyzed to Ran-GDP (Krichevsky et al., 2006). The exported molecule is released into the cytoplasm, XPO1 is recycled back to the nucleus and Ran-GDP is directed to the nuclear import cycle (Meier, 2005). Although the XPO1-dependent nucleocytoplasmic shuttling is the best-characterized nuclear export pathway, XPO1-independent pathways have been proposed for the nuclear export of various proteins. For example, the nuclear export of the African swine fever virus p37 protein is mediated by both the XPO1-dependent and XPO1-independent nuclear export pathways. Two signals responsible for the XPO1-mediated nuclear export of p37 protein were identified at the N terminus of the protein, and an additional signal was identified at the C-terminal region, which mediates the XPO1-independent nuclear export (Eulálio et al., 2006).

The nucleocytoplasmic shuttling of viral proteins is implicated in virus infections of metazoans (Weidman et al., 2003). The phosphoprotein (P) of human parainfluenza virus type 2 containing a functional NLS and an XPO1-dependent NES is a nucleocytoplasmic shuttling protein and its nucleocytoplasmic transport appears important for efficient viral polymerase activity (Ohtsuka et al., 2019). Many plant virus proteins also contain NLS and NES motifs that usually play multiple functional roles during infection (Krichevsky et al., 2006). For example, the N-terminal basic amino acid cluster _4_KRNKGGKKSR_13_ in the beet black scorch virus (BBSV) capsid protein (CP) is essential for its nuclear localization. The BBSV CP interacts with the nuclear import factor importin α, raising the possibility that the nuclear import of CP is mediated by importin α (Zhang et al., 2011). It has been postulated that nuclear-cytosolic transport pathways may be exploited by diverse viruses to facilitate replication and systemic infection (Weidman et al., 2003; Krichevsky et al., 2006). For instance, the monopartite geminivirus, tomato yellow leaf curl Java virus (ToLCJAV) encodes the nuclear shuttle protein V1, which could transport the genomic DNA between the nucleoplasm and the cytoplasm (Sharma and Ikegami, 2009). The P20 protein of bamboo mosaic potexvirus satRNA (satBaMV) interacts with the nucleolar protein fibrillarin and the interaction prompts the movement of satBaMV via the fibrillarin-satBaMV-P20 ribonucleoprotein complex in phloem-mediated systemic trafficking (Chang et al., 2016).

Potyviruses adopt polyprotein processing and RNA polymerase slippage-derived viral subpopulation as their genome expression strategy (Yang et al., 2021). Among the 11 known viral proteins, the nuclear inclusion protein b (NIb) is a well-established nuclear-located protein and contains the RNA-dependent RNA polymerase domain absolutely required for viral genome replication (Shen et al., 2020). Potyviral replication is a complex and sophisticated process that occurs in cytoplasmic membrane-bound virus replication complexes (VRCs), which consists of several viral proteins such as NIb and many host factors. To date, many nucleus-localized viral proteins and host factors have been also identified to play essential functional roles in VRCs of other plant RNA viruses, and interestingly, these VRCs are often present in dense masses adjacent to the nucleus (Krichevsky et al., 2006; Nagy and Pogany, 2011; Wang, 2015). Recently, we have shown that XPO1 is involved in the formation of the VRCs of TuMV by interacting with NIb to facilitate nuclear export of viral and host proteins to the VRCs and promote viral RNA replication (Zhang et al., 2021). In this study, we further identified the functional NLS and NES motifs of NIb, which enable NIb shuttling between the nucleus and the cytoplasm. We found that the NLS and NES motifs of NIb could directly bind to the N-terminal importin-beta and C-terminal CRM1 domain of NbXPO1a to regulate the nuclear import and export of NIb and viral RNA accumulation. Our data suggest that XPO1 has an important role in the nucleocytoplasmic transport of NIb by recognizing its NLS and NES to support potyviral infection.

## Results

### Functional Analysis of the Putative Nuclear Export Signal of NIb

**FIGURE 1:**
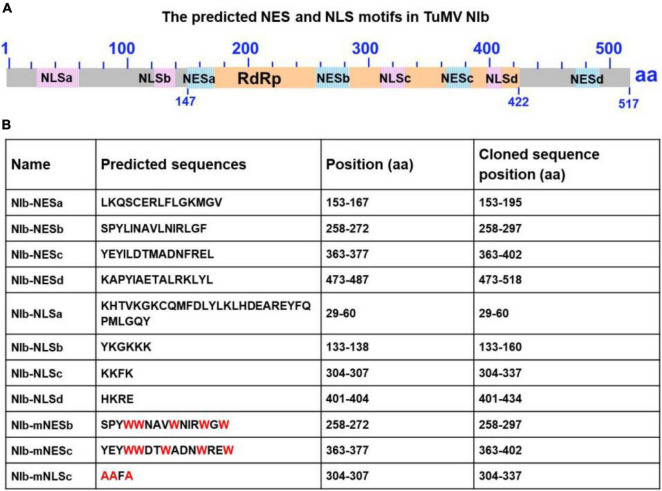
Sequence analysis of the turnip mosaic virus (TuMV) NIb protein. **(A)** Predicted TuMV NIb nuclear export signal (NES) and nuclear import signal (NLS) motifs are indicated by blue boxes and pink boxes in the NIb sequence. The RNA-dependent RNA polymerase (RdRP) domain is highlighted by the yellow box. **(B)** Detailed sequence information of the putative NIb NES and NLS motifs. For the mutation of NESs, mNESb or mNESc, tryptophan (Trp, W) was instead of Φ (L,I,V,M, or F) (These mutations are indicated in red). For the mNLSc, the three basic amino acids lysine (K) were replaced with alanine (A) (red).

**FIGURE 2:**
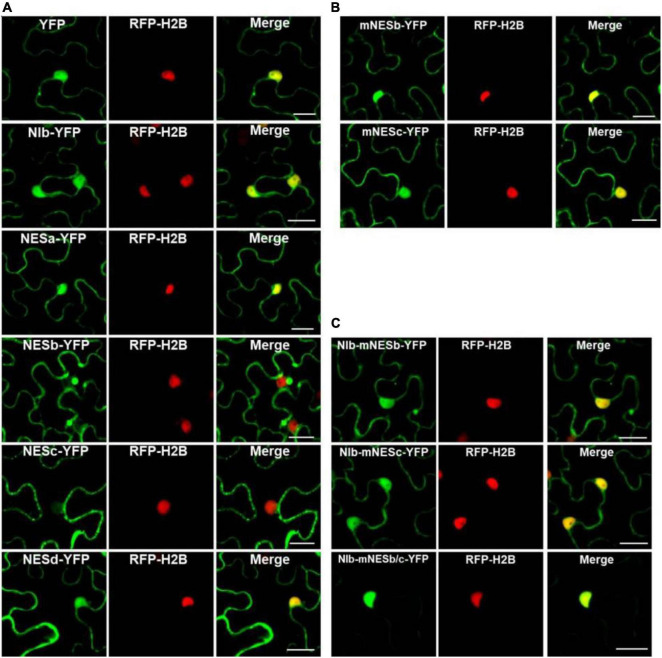
Analyses of the putative NESs of NIb. **(A,B)** Localization of the four putative NIb NESs. Four predicted sequences of the NIb NES (NESa, NESb, NESc, and NESd) domains and the mutated NESb (mNESb) and NESc (mNESc) sequences (Figure 1B) were fused to the YFP tagged vector. *Agrobacterium* cultures carrying YFP (as a control), NESa-YFP, NESb-YFP, NESc-YFP, NESd-YFP, mNESb-YFP or mNESc-YFP and NIb-YFP were infiltrated onto RFP-H2B (as a nuclear marker, red) transgenic *Nicotiana benthamiana* plants, and fluorescence (green) was visualized at 32 h post infiltration (hpi). YFP and NIb-YFP were distributed in both the nucleus and cytoplasm, and the putative NESa and NESd that fused with YFP had subcellular localizations similar to YFP. The putative NESb and NESc domains that fused YFP were able to export YFP from the nucleus to the cytoplasm, but the mutated NESb and NESc domains failed to function in nuclear export. **(C)** Localization of NIb carrying the NESb mutation (NIb-mNESb), NESc mutation (NIb-mNESc), or NESb and NESc (NIb-mNESb/c). These full-length NIb mutants were fused to the YFP tagged vector and fluorescence (green) was examined at 32 hpi. The single NES mutation on NIb-YFP failed to alter the fluorescence distribution, but the double NES mutations on NIb-YFP lost its nuclear export activity. Images represent single plain micrographs and bars = 25 μm **(A–C)**.

To characterize the nuclear export activity of TuMV NIb, we employed ‘‘LocNES’’^1^ and ‘‘NESmapper’’^2^ to predict its potential NES (Kosugi et al., 2014; Xu et al., 2015). The analysis resulted in the identification of four putative NIb NES motifs, and the four putative NIb NES motifs were named NESa-d (Figure 1). We then cloned each of them into the vector that contains YFP tag and conducted an agroinfiltration-mediated transient expression assay in the transgenic *Nicotiana benthamiana* plants that stably express red fluorescent proteins fused to histone 2B (RFP-H2B) to evaluate their nuclear export ability (Martin et al., 2009; Anderson et al., 2018). Upon translation, RFP-H2B targets the nucleus and serves as a nuclear marker. The estimated molecular weight of NES (a,b,c and d)-YFP fusions is 36.47, 36.32, 36.5, 36.8 KDa, respectively, and protein expression of the above fused constructs was confirmed by Western blot (Supplementary Figure 1). The fluorescence (green) emitted by NES-YFP fusion proteins was visualized under a confocal microscope at 32 h post infiltration (hpi). Since NPC permits passage of ions and small molecules (usually 20 to 30 kDa) with a maximal size of ∼60 kDa (Nigg, 1997; Eibauer et al., 2015), YFP (∼26 kDa) alone (which does not contain any known NLS or NES) was distributed in both the nucleus and the cytoplasm (Figure 2A). NIb-YFP (∼84 kDa) containing potential NES and NLS was also distributed in both the nucleus and cytoplasm (Figure 2A), suggesting active nuclear-cytoplasmic transport does exist for NIb. NESa- and NESd- YFP fusions were also found in the nucleus and cytoplasm. In contrast, NESb- and NESc- YFP fusions were predominantly present in the cytoplasm and did not accumulate in the nucleus. Based on these data, we conclude that the putative NIb NESb and NESc are authentic NESs.

It is known that the proteins are produced in the cytoplasm, and many small proteins (usually 20–30 kDa) are often incorporated in the nucleus, and afterward they are translocated back to the cytoplasm under the guidance of NES. To demonstrate whether NESb- and NESc-YFP fusions do also enter the nucleus and then shuttle back to the cytoplasm, we applied the inhibitor of nuclear export, leptomycin B (LMB) (Sun et al., 2013), to analyze the dynamic subcellular localization of NESb-YFP and NESc-YFP. The treatment of LMB inhibited the nuclear export of NESb- and NESc-YFP (Supplementary Figures 2A,B) and significantly increased the fluorescence intensity of NESb-YFP and NESc-YFP in the nucleus (Supplementary Figure 2C), confirming the nuclear export function of NES2 and NES3.

The putative NESb sequence of TuMV NIb is consistent with the “classical NES consensus amino acid sequence”: Φ–X2,3–Φ–X2,3–Φ–X–Φ, where Φ represents leucine (Leu, L), isoleucine (Ile, I), valine (Val, V), methionine (Met, M), or phenylalanine (Phe, F), and X2,3 represents any two or three amino acids (Dong et al., 2009). The putative NESc sequence of TuMV NIb is consistent with the “Class 3 NESs pattern” (Kosugi et al., 2008): Φ–X2–Φ–X3–Φ–X2–Φ (Φ: L, I, V, M, or F, X2: any two amino acids, X3: any three amino acids). We created point mutations to further characterize the nuclear export function of NESb and NESc. Mutants mNESb and mNESc were generated to substitute the residue Φ (L,I,V,M, or F) with tryptophan (Trp, W) (Figure 2B) and the resulting mutants were cloned to the vector that contains YFP tag. Confocal microscopy data showed that mNESb or mNESc lost the ability to be exported from the nucleus. Furthermore, full-length NIb derivatives harboring the corresponding NESb or NESc mutations in which residue Φ (L,I,V,M, or F) are replaced by tryptophan (Trp, W) (named NIb-mNESb or NIb-mNESc, respectively), or double mutations of NESb and NESc (NIb-mNESb/c) were engineered by overlapping PCR and specific primers (Supplementary Table 1). These NIb derivatives were then cloned into a YFP vector to enable fluorescence observations of their subcellular localization. As shown in Figure 2C, NIb-mNESb-YFP and NIb-mNESc-YFP localized in the nucleus and cytoplasm, similar to NIb-YFP, which indicated NESb and NESc can function independently. In contrast, NIb-mNESb/c-YFP only localized in the nucleus, supporting that NIb contains two functional NESs.

### Functional Analysis of the Putative Nuclear Localization Signal Motifs of NIb

**FIGURE 3:**
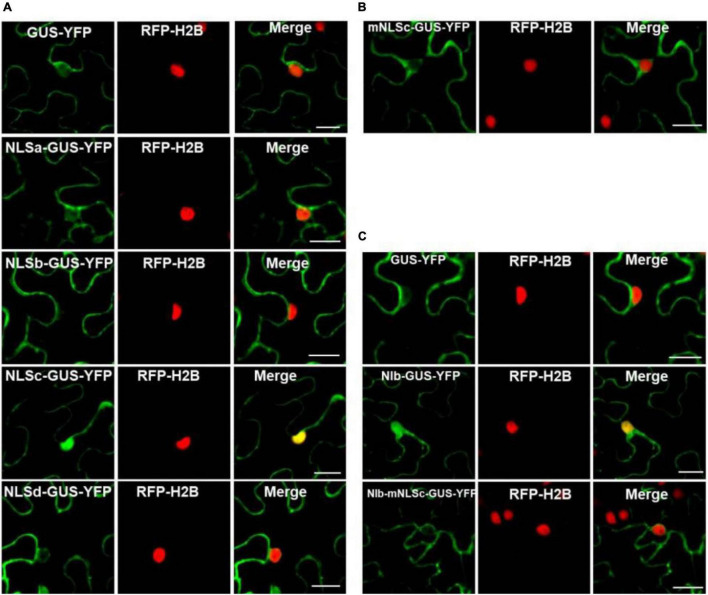
Analyses of the putative NLSs of NIb. **(A,B)** Localization of the putative NIb NLSs. Four putative NIb NLS (NLSa, NLSb, NLSc and NLSd) sequences and the mutated NLSc (mNLSc) were fused to the GUS-YFP vector. *Agrobacterium* cultures carrying the GUS-YFP (as a control), Green fluorescence shows the localization of each protein, and fluorescence was examined at 32 hpi. **(C)** Localization of NIb and the NIb carrying NLS mutation (NIb-mNLS) fused to GUS-YFP fluorescence at 32 hpi. RFP-H2B as a nucleus marker (red). Images represent single plain micrographs and bars = 25 μm **(A–C)**.

The NIb NLS motifs were predicted by ‘‘cNLS Mapper’’^3^ and ‘‘NLStradamus’’^4^ (Kosugi et al., 2009; Nguyen Ba et al., 2009), and the four potential NLSs (NLSa-d) were obtained (Figure 1). To assess these putative NLSs, we constructed a plant expression vector to express β-glucuronidase (GUS) tagged by YFP (GUS-YFP) (∼93 kDa). As expected, GUS-YFP alone failed to localize in the nucleus because either GUS or YFP sequence lacks an efficient NLS (Figure 3A). Each of the four putative NLS motifs was cloned into the GUS-YFP vector for transient expression in RFP-H2B transgenic *N. benthamiana* plants and subsequent confocal microscopy analyses of nuclear import activity at 32 hpi. The GUS-YFP vector, NLSa-GUS-YFP, NLSb-GUS-YFP and NLSd-GUS-YFP all localized predominantly in the cytoplasm whereas NLSc-GUS-YFP accumulated clearly in the nucleus and cytoplasm (Figure 3A). These data suggest that the putative NLSa, NLSb or NLSd domain alone is not sufficient to exert the NLS function and the putative NLSc alone has strong nuclear import activity.

Classical NLSs are a short, basic amino acid-rich sequence that includes two types of NLSs. One type NLS includes 3–5 consecutive basic amino acid residues, as to K-K/R-X-K/R (K, R represents lysine, arginine respectively, X represents any amino acid), which is called monopartite NLS. The other type NLS consists of K/RK/R-X_10–12_-K/R_3/5_ (3/5 represents that three of the five consecutive amino acids are lysine or arginine), which is called bipartite NLS (Lange et al., 2007; Xu et al., 2010). The putative NLSc sequence of TuMV NIb (KKFK) is consistent with the monopartite NLS: K-K/R-X-K/R, where X represents phenylalanine (Phe, F). We further generated an NLSc mutant for a lost-function assay. Substitution of the three basic lysines (K) in NLSc with alanines (A) abolished its nuclear localization capacity (Figure 3B), further supporting that NIb NLSc is an authentic NLS. The expression of NLSa-GUS-YFP, NLSb-GUS-YFP, NLSc-GUS-YFP, NLSd-GUS-YFP, and mNLSc-GUS-YFP was confirmed by Western blot assay (Supplementary Figure 3). Consistently, the full-length NIb derivative harboring the NLS mutations (NIb-mNLSc) only accumulated in the cytoplasm, in contrast to the nuclear and cytoplasm localization of NIb-GUS-YFP (Figure 3C). Therefore, we conclude that NIb has only one functional NLS.

### Mapping the Nuclear Export Signals and Nuclear Localization Signal of NIb and Its Interaction Motif With XPO1

**FIGURE 4:**
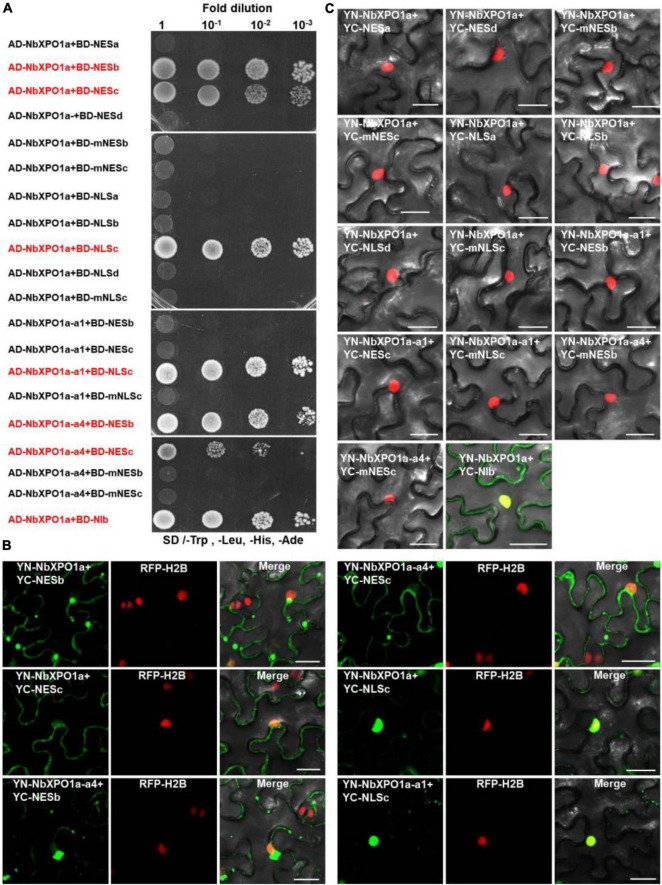
NbXPO1a interacts with NIb NES and NLS domains. **(A)** Y2H assays to detect interactions between NbXPO1a, the a1 and a4 fragments of NbXPO1a with the NIb NES and NLS domains and their respective mutant sequences. NbXPO1a-a1 and NbXPO1a-a4 are two NbXPO1a deletion mutants that contain the IBN_N domain and the CRM1_C domain, respectively. Y2H Gold yeast cells harboring the indicated plasmids were co-expressed, subjected to 10-fold serial dilutions and plated on synthetic dextrose (SD)/-Trp, -Leu, -His, -Ade agar to identify protein interactions. **(B,C)** BiFC assays in RFP-H2B transgenic *N. benthamiana* (red) leaves at 32 hpi. Green fluorescence was observed as a consequence of the complementation of YN tagged NbXPO1a, and the a1 (NbXPO1a-a1) and a4 (NbXPO1a-a4) fragments of NbXPO1a with the YC tagged NIb NES or NLS sequences and their respective mutants shown in Figure 1B. The yellow fluorescence results from overlapping of the green BiFC and the red RFP-H2B fluorescence. Bars = 25 μm.

Our recent study showed that the N-terminal importin-beta (IBN_N) and the C-terminal CRM1 (CRM1_C) domain of XPO1 interacts with TuMV NIb (Zhang et al., 2021). To map the interaction motifs of NIb and XPO1, we performed yeast two-hybrid (Y2H) and bimolecular fluorescence complementation (BiFC) assays. The four putative NIb NESs, the four putative NIb NLSs and their mutants were examined for their possible interactions with NbXPO1a and two NbXPO1a truncated mutants NbXPO1a-a1 and NbXPO1a-a4 that contain the IBN_N domain and the CRM1_C domain of NbXPO1a, respectively. We found that NbXPO1a and NbXPO1a-a4 bound to NIb by interacting with one of its two NESs (NESb and NESc) but not with the NESa, NESd, mNESb or mNESc of NIb in the cytoplasm (Figures 4A,B). In the nucleus, NbXPO1a and NbXPO1a-a1 bound to the NIb NLSc but not to the NLSa, NLSb, NESd, mNLSc of NIb (Figures 4A,B). These assays revealed that the CRM1_C domain of NbXPO1a (NbXPO1a-a4) bound to the NIb NESb and NESc motifs and that the interacting pairs localized in the cytoplasm. However, only NbXPO1a and its a1 fragment including the IBN_N domain (NbXPO1a-a1) were associated with NIb NLSc in the nucleus. Together these results suggest that the two functional NESs and one functional NLS in NIb are responsible for nuclear export and import of NIb by binding to the CRM1_C domain and IBN_N domain of XPO1, respectively.

### The Mutation of Functional Nuclear Export Signals and Nuclear Localization Signal of NIb Impairs Viral Infection

**FIGURE 5:**
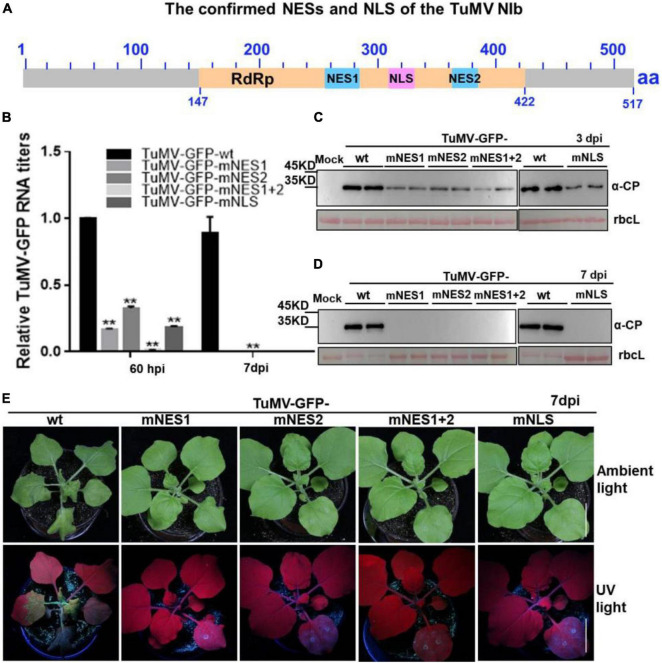
The mutation of NESs and NLS of NIb impairs viral infection. **(A)** The confirmed NESs and NLS in this study were renamed and noted by solid blue boxes and solid pink box, respectively. RdRP, RNA-dependent RNA polymerase, was noted by yellow box. **(B–D)** The wild type (wt) TuMV-GFP infection clone, and the TuMV-GFP infection clone harboring the mutation of functional NES1, NES2, NES1 and NES2 (NES1/NES2), or NLS were infiltrated onto *N. benthamiana* leaves. At 60 hpi and 7 days post infiltration (dpi), total RNA and protein were extracted from the infiltrated leaves and systemic leaves for RT-qPCR analysis and western blot analysis **(B–D)**. Values represent means relative to the leaves infiltrated with TuMV plus Mock. Each average value was calculated by three representative replicates. The data were analyzed using Student’s *t* test and double asterisks indicate *P* < 0.01. *NbActin* was used as an internal control. The large Rubisco subunit (rbcL) staining with Ponceau S showed the equal loading. **(E)** Viral symptoms and GFP fluorescence in plants inoculated with the indicated infectious clones were photographed under natural and UV light at 7 dpi.

Based on the above findings, we renamed the putative NESb and NESc to functional NES1 and NES2, and the putative NLSc to functional NLS, respectively (Figure 5A). We introduced the above mutations of functional NES1, NES2, NES1 + NES2 and NLS into a TuMV-GFP infectious clone. The resulting TuMV-GFP mutants were agroinfiltrated onto *N. benthamiana* leaves, and the accumulation of viral RNA and protein was then subjected to RT-qPCR and immunoblotting analyses. At 60 hpi, in the plants agroinfiltrated with the mutant clones harboring the mutation of functional NES1, NES2, NLS, or NES1 and NES2 (mNES1 + 2) of NIb, viral RNA and protein accumulation were significantly inhibited compared to the wild type TuMV-GFP infectious clone (Figures 5B,C). Moreover, at 7 days post infiltration (dpi) and 20 dpi, the plants agroinfiltrated with the TuMV mutants did not display any obvious viral symptoms, and no viral RNA and protein accumulation was detectable in the systemic leaves of these plants (Figures 5B,D,E and Supplementary Figure 4). These data indicated that the functional NES1, NES2 and NLS of NIb are important for viral RNA accumulation and systemic infection.

### The Interaction Domains of XPO1 With the Nuclear Export Signals and Nuclear Localization Signal of NIb Promote Viral RNA Accumulation and Infection

**FIGURE 6:**
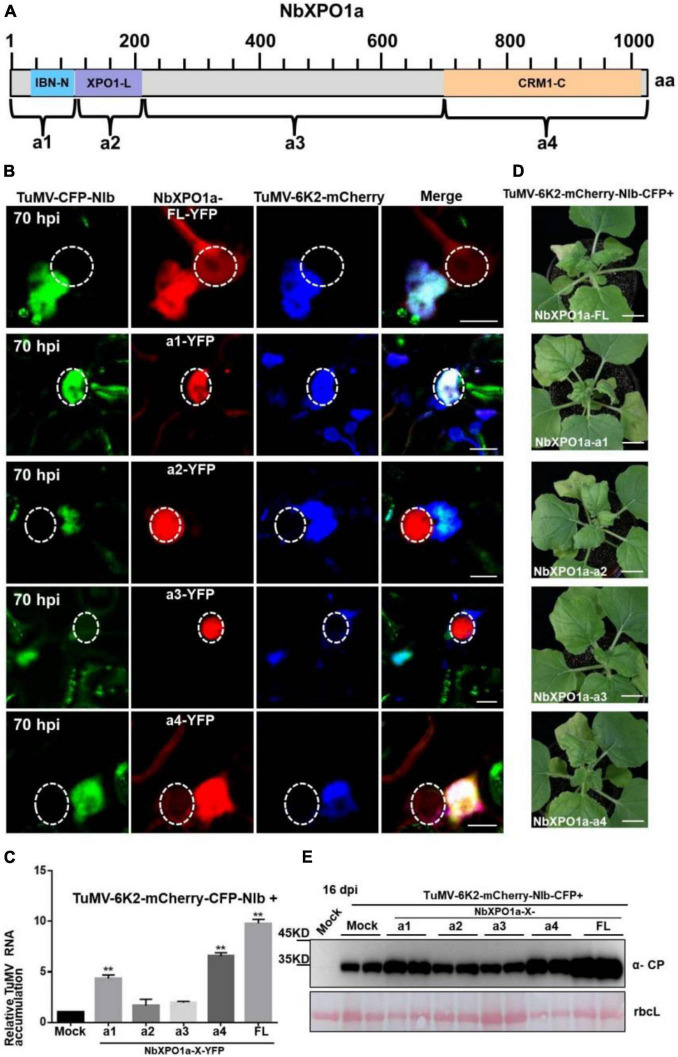
Overexpression of the IBN_Nand CRM1_C domain of NbXPO1a facilitate viral accumulation and systemic infection. **(A)** Schematic representation of full-length NbXPO1a and four NbXPO1a fragments. **(B)** Transiently expressed YFP fusions with full-length NbXPO1a-YFP (FL), NbXPO1a-a1-YFP (a1), NbXPO1a-a2-YFP (a2), NbXPO1a-a3-YFP (a3), or NbXPO1a-a4-YFP (a4) in *N. benthamiana* leaf cells infected with TuMV-6K2-mCherry-CFP-NIb at 70 hpi. Images represent single plain micrographs and bars = 10 μm. The region of nucleus is highlighted with a white circle. **(C)** RT-qPCR analyzed TuMV RNA levels. RNA was extracted from the infiltrated leaves as indicated in panel **(A)**. Values represent means relative to the leaves infiltrated with TuMV-6K2-mCherry-CFP-NIb plus Mock. And each average value was calculated by three representative replicates. The data were analyzed using Student’s *t* test and double asterisks indicate *P* < 0.01. *NbActin* was used as an internal control. **(D)** Viral symptoms of TuMV-6K2-mCherry-CFP-NIb-infected *N. benthamiana* plants at 16 dpi. Transient overexpression of the full-length NbXPO1a and truncated NbXPO1a fragments in leaf cells infected with TuMV-6K2-mCherry-CFP-NIb. Bars = 2 cm. **(E)** Western blot analysis of viral protein accumulation. Total protein was extracted from the systemic leaves indicated in panel **(C)**. Anti-TuMV CP antibodies were used, and the large Rubisco subunit (rbcL) staining with Ponceau S showed the equal loading. Mock: *N. benthamiana* plant was infiltrated with *Agrobacterium tumefaciens* culture carrying the empty vector.

NbXPO1a interacts with NIb NESs and NLSs by its IBN_N domain, and CRM1_C domain, which promoted us to further investigate their functions in the context of the formation of VRCs, viral RNA accumulation and systemic infection. We conducted an agroinfiltration assay in *N. benthamiana* plants to transiently co-express the viral infectious clone TuMV-6K2-mCherry-CFP-NIb that encodes 6K2-mCherry and CFP-tagged NIb (Cheng et al., 2015) and the full-length NbXPO1a (FL) or each of the four truncated NbXPO1a including a1 (the IBN_N domain), a2 (the exportin 1-like domain, XPO1-L), a3 (an unknown function fragment downstream of a2) and a4 (the CRM1_C domain). NbXPO1a and the truncated fragments were fused with YFP at their C-terminals (Figure 6A). The infiltrated leaves were examined for confocal analysis, and microscope images were captured at 70 hpi. It was obvious that NbXPO1a-YFP co-localized with the VRCs which were associated with CFP-NIb- and 6K2-mCherry-stained aggregates shown as irregular structures around the nuclear periphery (Figure 6B). NbXPO1a-a1-YFP primarily co-localized with CFP-NIb in the nucleus, NbXPO1a-a4-YFP co-localized with CFP-NIb at the periphery of the nucleus, while no obvious co-localization was found between NbXPO1a-a2-YFP or NbXPO1a-a3-YFP with CFP-NIb. The transient over-expression of NbXPO1a-a1 and NbXPO1a-a4 increased TuMV-6K2-mCherry-CFP-NIb RNA accumulation at 70 hpi (Figure 6C). Consistently, transient over-expression of NbXPO1a, NbXPO1a-a1 and NbXPO1a-a4 also promoted the systemic infection and the protein accumulation of TuMV-6K2-mCherry-CFP-NIb at 16 dpi (Figures 6D,E). These data suggest that the IBN_N domain and the CRM1_C domain of NbXPO1a facilitate viral accumulation and systemic infection.

## Discussion

The potyviral RNA polymerase, NIb, is a nuclear targeting protein (Restrepo et al., 1990; Li et al., 1997; Revers and García, 2015). We functionally identified that the TuMV NIb protein contains one strong NLS and two NESs in the middle domain (Figures 1–3) that mediate the nucleocytoplasmic shuttling of NIb. A previous report showed that the NPC size exclusion limit is far larger than 60 kDa and depends on the structure, charges and hydrophobicity of transported molecules (Wang and Brattain, 2007). However, numerous smaller proteins are regulated via active mechanisms during nuclear entry or exit, rather than by passive diffusion through the NPC channel (Rajamäki and Valkonen, 2009). NIb is approximately 60 kDa, but its nuclear-cytosolic shuttling depends on both viral proteins and host receptors (Figures 1–4; Zhang et al., 2021). The functional NESs and NLS of NIb are located in the RdRP domain, which is the most conservative structural domain of viral RdRPs containing the palm subdomain (Bruenn, 2003; Ferrer-Orta et al., 2015). This subdomain is comprised of five structural conservative motifs that are extremely important for viral RNA replication, such as the GDD sequence motif that is essential for RdRP activity and is a hallmark of viral RdRPs (De Farias et al., 2017; Shen et al., 2020). Of note, the TuMV NIb protein contains two functional NESs. Mutation of one of them did not affect the nuclear export of NIb, whereas mutation of both of them abolished the nuclear export capability of NIb (Figure 2). However, their individual mutation on the viral infections clone inhibited viral RNA accumulation and impaired viral systemic infection (Figures 2–4). These data demonstrate that the functional roles of the NES and NLS of NIb are not just limited to the NIb nucleocytoplasmic shuttling.

NbXPO1a contains four domains/fragments, namely (from the N-terminus), the IBN_N domain, the exportin 1-like protein (XPO1-L) domain, a fragment without any putative function and the CRM1_C domain (Zhang et al., 2021). Our previous study showed that the IBN_N and CRM1_C domains of XPO1 interact with TuMV NIb to facilitate viral infection. Here we further analyzed the interactions of NIb NESs and NLSs with XPO1 and its N-terminal and C-terminal domain. NIb NESs were found to bind to the CRM1 domain at the nuclear periphery. This finding was consistent with the fact that XPO1 mediates the nuclear export of target proteins that contain a leucine-rich NES (Fornerod et al., 1997; Fukuda et al., 1997; Ossareh-Nazari et al., 1997). Interestingly, we found that the NIb NLS bound to the IBN_N domain of XPO1 in the nucleus (Figures 3, 6). Thus, the nuclear import of NIb is mediated likely via the interaction between the NIb NLS and the XPO1 IBN_N domain. Consistently, the mutated NIb NLS that failed to localize in the nucleus also lost the ability to interact with the XPO1a IBN_N domain (Figures 3, 4). This result reveals that XPO1 has the nuclear import activity that is potentiated by the XPO1 IBN_N domain. Furthermore, over-expression of the IBN_N domain and CRM1_C domain of XPO1a co-localized with TuMV CFP-NIb in the nucleus and at the VRCs, respectively, and their expression increased viral RNA accumulation and systemic infection (Figure 6). These findings suggest that XPO1 could regulate NIb nuclear-cytosolic shuttling by its IBN domain-mediated nuclear import and its CRM1 domain-mediated nuclear export.

## Materials and Methods

### Plant Materials and Growth Conditions

*Nicotiana benthamiana* were potted in sandy loam soil and placed in an insect-free growth chamber. The growth conditions consisted of 60% relative humidity and a day/night regime of 16 h in the light at 22°C followed by 8 h of darkness at 18°C. The transgenic RFP-H2B line was a gift of Dr. Michael M. Goodin (University of Kentucky, United States).

### Information on Turnip Mosaic Virus and Its Infectious Clones

The NIb coding sequences of TuMV (nucleotides range: 7208–8758 nt; GenBank accession: NC002509) was used for the informatics and experimental analyses. TuMV-GFP infectious construct contains a GFP coding sequence that was inserted in between P1 and HC-Pro coding regions, TuMV-6K2-mCherry-CFP-NIb encodes 6K2-mCherry that was inserted in between P1 and HC-Pro coding regions and cyan fluorescent protein (CFP)-tagged NIb (Thivierge et al., 2008; Cotton et al., 2009; Cheng et al., 2017).

### Plasmid Construction

Y2H constructs: AD or BD vector carrying TuMV NIb or its NLSs or NESs, NbXPO1a (Gene accession: MK935565) and its four fragments, and BiFC constructs: YN or YC carrying TuMV NIb or its NLSs or NESs, NbXPO1a and its four fragments have been described previously (Li et al., 2018; Zhang et al., 2021). Four putative NIb NESs, NLSs and their mutants were obtained by PCR using primers list in Supplementary Table 1. The full-length NIb derivatives harboring the NESb (NIb-mNESb), NESc (NIb-mNESc), or both the NESb and NESc (NIb-mNESb/c), or NLSc (NIb-mNLSc) mutations were engineered by overlapping PCR and specific primers (Supplementary Table 1). The resulting DNA fragments were purified and transferred into the entry vector pDONR221 (Invitrogen) by recombination using BP Clonase^®^ (Invitrogen). Insertions in the resulting pDONR clones were verified by DNA sequencing and of the resulting intermediate pDONR221 cloned inserts were transferred into modified Gateway-compatible vectors including Y2H vectors, BiFC vectors and other transient expression vectors (Earley et al., 2006; Lu et al., 2010; Li et al., 2018).

For the generation of the mutated infectious clone TuMV carrying the mutation of NES1, NES2, NLS, or NES1 and NES2 (mNES1 + 2) of NIb (these NESs and NLS have been renamed based on their nuclear export and import activity), three round reactions of overlapping PCR were conducted using specific primers (Supplementary Table 1) and the fused PCR products were digested with restriction endonuclease *Avr*II. The digested fused PCR products were re-ligased into the pCambia2300-TuMV-GFP backbone vector in which the original fragment was removed by digestion of the same restriction enzymes. The recombinant plasmids were further confirmed by sequencing.

### Agroinfiltration and Viral Inoculation

Wild type or RFP-H2B transgenic *N. benthamiana* plants at the 4 to 5 leaf stage were used for *Agrobacterium-*mediated transient expression of the recombinant binary constructs. The details of procedures as described (Li et al., 2017; Zhang et al., 2021). For TuMV infection analysis, *Agrobacterium* cultures carrying the wild type (wt) TuMV-GFP infection clone (OD_600_ = 0.5), and the TuMV-GFP infection clone harboring the mutation of NES1, NES2, NES1 and NES2 (NES1/NES2), or NLS (OD_600_ = 0.5, respectively) were infiltrated onto *N. benthamiana* leaves. Inoculated plants were photographed with a Canon 400D digital camera at various times.

### RNA Extraction and RT-qPCR Analysis

Total RNA was extracted from *N. benthamiana* leaves with the RNeasy Plant Mini Kit and treated with DNase I as instructed by the manufacturer (Qiagen). Synthesis of cDNA and RT-qPCR were as described previously (Li et al., 2014). Primers used for RT-PCR are shown in Supplementary Table 1, and the specific primer pairs (Supplementary Table 1) used for RT-qPCR were designed by Primer Premier 5 software. RT-qPCR reactions were conducted and analyzed as described previously (Li et al., 2014).

### Immunoblotting

TuMV-GFP infectious and its mutants that harbored the mutation of NES1, NES2, NLS, or NES1 and NES2 (mNES1 + 2) of NIb were infiltrated onto *N. benthamiana* leaves. The infiltrated leaves at 60 hpi and the newly emerged leaves at 7 dpi were collected for total protein extraction. Immunoblotting was performed with mouse monoclonal antibodies: anti-CP antibodies (1:10000). Immunoblotting of NESa-YFP, NESb-YFP, NESc-YFP, NESd-YFP, mNESb-YFP, mNESc-YFP, NLSa-YFP, NLSb-YFP, NLSc-YFP, NLSd-YFP, and mNLSc-YFP was performed with primary antibodies: anti-GFP polyclonal antibodies (1:5000; catalog number: 11814460001, Roche). Above reactions were followed by incubating with goat anti-mouse secondary antibodies obtained from Esaybio (1:5000, catalog number BE02) conjugated to horseradish peroxidase. Blotted membranes were washed thoroughly and chemiluminescence was visualized as described by the manufacturer’s protocol (ECL; GE Healthcare).

## Data Availability Statement

The original contributions presented in the study are included in the article/Supplementary Material, further inquiries can be directed to the corresponding authors.

## Author Contributions

FL designed the project. MZ, PG, LG, YL, ZC, and RQ conducted the experiments. FL, AW, and XZ wrote the manuscript. All authors analyzed the data and reviewed the manuscript.

## Conflict of Interest

The authors declare that the research was conducted in the absence of any commercial or financial relationships that could be construed as a potential conflict of interest.

## Publisher’s Note

All claims expressed in this article are solely those of the authors and do not necessarily represent those of their affiliated organizations, or those of the publisher, the editors and the reviewers. Any product that may be evaluated in this article, or claim that may be made by its manufacturer, is not guaranteed or endorsed by the publisher.

## Footnotes

1
http://prodata.swmed.edu/LocNES/LocNES.php

2
http://sourceforge.net/projects/nesmapper

3
http://nls-mapper.iab.keio.ac.jp/cgi-bin/NLS_Mapper_form.cgi

4
http://www.moseslab.csb.utoronto.ca/NLStradamus/
