# Supplementary material for: Nuclear Exportin 1 (XPO1) Binds to the Nuclear Localization/Export Signal of the Turnip Mosaic Virus NIb to Promote Viral Infection
Source: Front Microbiol. 2022 Jan 4;12:780724. doi: 10.3389/fmicb.2021.780724 (PMC8763854; doi:10.3389/fmicb.2021.780724)
Supplement: Supplementary file 1 [file Data_Sheet_1.pdf]

Supplementary Information

Supplementary Table1

TuMV-CP-q-F	TGGCTGATTACGAACTGACG
TuMV-CP-q-R	CTGCCTAAATGTGGGTTTGG
NbActin2-qPCR-F	AAAGACCAGCTCATCCGTGGAGAA
NbActin2-qPCR-R	TGTGGTTTCATGAATGCCAGCAGC
NbXPO1a-gw-F	GGGGACAAGTTTGTACAAAAAAGCAGGCTTCATGGCGGCGGAGAAGCTTAGGG
NbXPO1a-gw-R	GGGGACCACTTTGTACAAGAAAGCTGGGTCTGAATCCACCATTTCGTCTTG
NbXPO1a-1-gw-R	GGGGACCACTTTGTACAAGAAAGCTGGGTCCTTTACAATAACTTCAGAAATG
NbXPO1a-2-gw-F	GGGGACAAGTTTGTACAAAAAAGCAGGCTTCATGCTTTCTAGTGATGAAGCATCACTT
NbXPO1a-2-gw-R	GGGGACCACTTTGTACAAGAAAGCTGGGTCTAAGCACTGAAGAGTGGGATTCC
NbXPO1a-3-gw-F	GGGGACAAGTTTGTACAAAAAAGCAGGCTTCATGACAGAGGTTGCTGTCCTCAATTTT
NbXPO1a-3-gw-R	GGGGACCACTTTGTACAAGAAAGCTGGGTCAGGCAAGAAATATGTACCAAG
NbXPO1a-4-gw-F	GGGGACAAGTTTGTACAAAAAAGCAGGCTTCATGCAAATTACTCTCATATTTCTG
NESa-F	GGGGACAAGTTTGTACAAAAAAGCAGGCTTCatgctgaaacaaagttgtgaacgg
NESa-R	GGGGACCACTTTGTACAAGAAAGCTGGGTCtagtggtgcggcagtaaacgtc
NESb-F	GGGGACAAGTTTGTACAAAAAAGCAGGCTTCatgtcgccatacttgatcaacgc
NESb-R	GGGGACCACTTTGTACAAGAAAGCTGGGTCtgtagaaataggggtatacacg
NESc-F	GGGGACAAGTTTGTACAAAAAAGCAGGCTTCatgtatgagtatattcttgacac
NESc-R	GGGGACCACTTTGTACAAGAAAGCTGGGTCtttgtgcccctggtgcgacataaac
NESd-F	GGGGACAAGTTTGTACAAAAAAGCAGGCTTCatgaaagctccttacatagcggaaacag

NESd-R	GGGGACCACTTTGTACAAGAAAGCTGGGTCctggtgataaacacaagcctcagc
NLSa-F	GGGGACAAGTTTGTACAAAAAAGCAGGCTTCatgaagcacacagttaaaggaaaatgt
NLSa-R	GGGGACCACTTTGTACAAGAAAGCTGGGTCatactggcccagcatcggttgg
NLSb-F	GGGGACAAGTTTGTACAAAAAAGCAGGCTTCatgggagcgttgtataaaggaaagaag
NLSb-R	GGGGACCACTTTGTACAAGAAAGCTGGGTCgagccgttcacaactttgtttc
NLSc-F	GGGGACAAGTTTGTACAAAAAAGCAGGCTTCatgaagaagttcaaaggaaacaatag
NLSc-R	GGGGACCACTTTGTACAAGAAAGCTGGGTCtttcttgagtgaatagttgactg
NLSd-F	GGGGACAAGTTTGTACAAAAAAGCAGGCTTCatgcacaaaagagagggaatctgg
NLSd-R	GGGGACCACTTTGTACAAGAAAGCTGGGTCtgcctctagtcgatggcatggc
mNESb-F	GGGGACAAGTTTGTACAAAAAAGCAGGCTTCatgtcgccatactGgTGGaacgcagtaTGGaacat
NIb-mNESb-overlap-F	tcgccatactGgTGGaacgcagtaTGGaacatccgctGGggatGGatggaagagtggg
NIb-mNESb-overlap-R	cccactcttccatCCatccCCagcggatgttCCAtactgcgttCCAcCagtatggcga
mNESc-F	GGGGACAAGTTTGTACAAAAAAGCAGGCTTCatgtatgagtatTGGTGGacactTGggcagacaactGGcgtgaaTGg
NIb-mNESc-F	tatgagtatTGGTGGacactTGggcagacaactGGcgtgaaTGggcctgaagtat
NIb-mNESc-R	atacttcaggcccCAttcacgCCagttgtctgcccCAagtgtcCCACCAatactcata
mNLSc-F	GGGGACAAGTTTGTACAAAAAAGCAGGCTTCatgGCgGCgttcGCaggaaacaatag
NIb-mNLSc-overlap-F	atggtacactcgtcGCgGCgttcGCaggaaacaatag
NIb-mNLSc-overlap-R	ctattgtttcctGCgaacGCcGCgacgagtgtaccat

Supplementary Figures

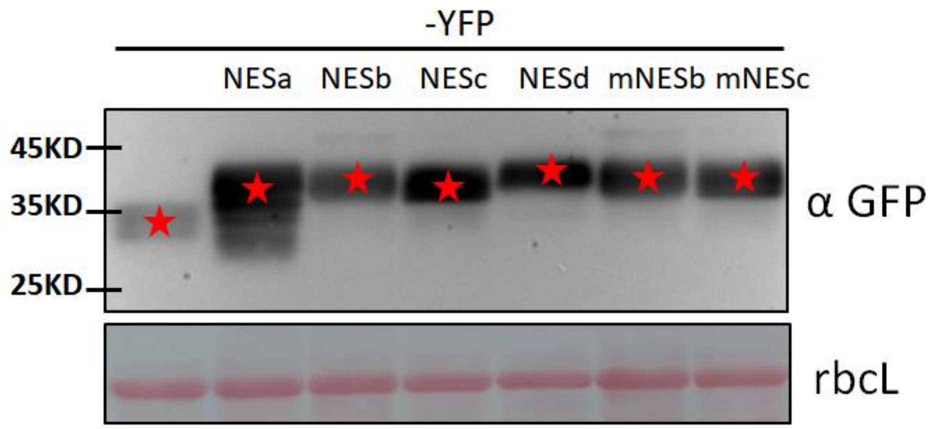

Fig S1. Confirmation of the expression of fusion proteins by immunoblotting. Western blot assay was used to test the expression of NESa, NESb, NESc, NESd, mNESb and mNESc of NIb fused with YFP. Total protein was extracted from the RFP-H2B *Nicotiana benthamiana* leaves expressing the plasmids indicated at 32 hours post infiltration (hpi). Antibody against GFP was applied (Note: anti-GFP antibody could recognize wild type GFP and mutant forms of GFP including YFP and CFP). The red star indicates the specific band and Ponceau staining of Rubisco large subunit (rbcL) serves as a loading control.

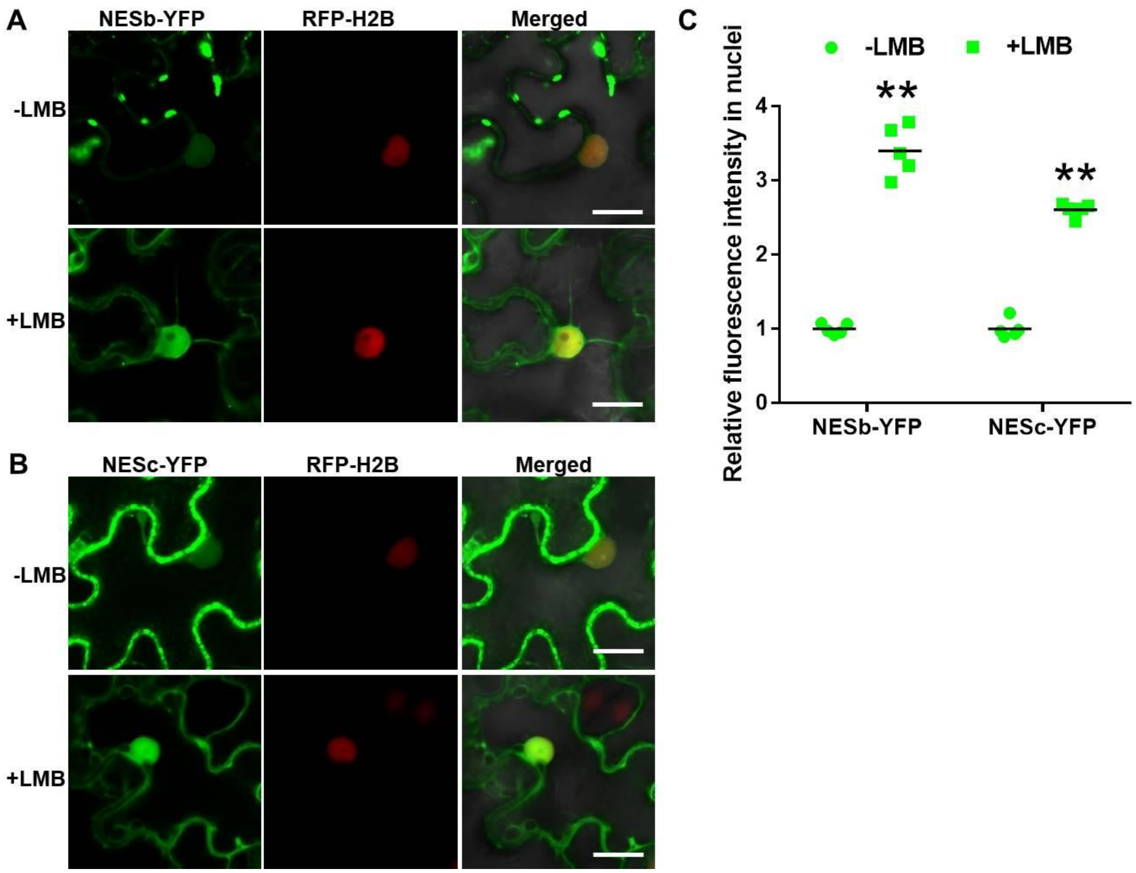

Fig S2. Verification of NIb NESs functions. (A-B) Subcellular localization of NESb-YFP and NESc-YFP without or with LMB treatment. RFP-H2B transgenic *N. benthamiana* leaves expressing NESb-YFP and NESc-YFP at 32 hpi were infiltrated with equal volumes of ddH_2O (-LMB) or 50 nM LMB (+LMB, LMB can be dissolved in ddH_2O), and the infiltrated regions were observed by confocal microscopy after 10 h (at 42 hpi). Images represent single plain micrographs and bars=25 μm. **(C)** Quantification of fluorescent signals in the nuclei of NESb-YFP or NESc-YFP with or without LMB treatment using Image J software. Values represent the fluorescence intensity of NESb-YFP or NESc-YFP in nuclei relative to -LMB treatment. Values are obtained by calculating 5 representative images. Double asterisks indicate highly significant differences (**P<0.01, Student's *t* test) compared to ddH2O (-LMB) treatment.

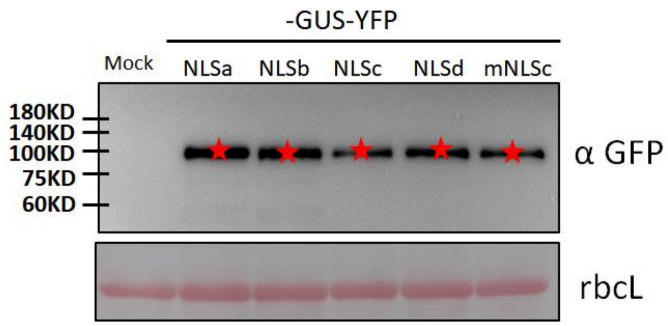

Fig S3. Confirmation of the expression of fusion proteins by immunoblotting. Protein expression of putative NLSa, NLSb, NLSc, NLSd, and mNLSc of NIb fused with GUS-YFP was confirmed by Western blot. Total protein was extracted from the RFP-H2B *N. benthamiana* leaves expressing the plasmids indicated. Mock: RFP-H2B *N. benthamiana* plant was infiltrated with *Agrobacterium tumefaciens* culture without the recombinant vector. Antibody against GFP was applied. The red star indicates the specific band and Ponceau staining of Rubisco large subunit (rbcL) serves as a loading control.

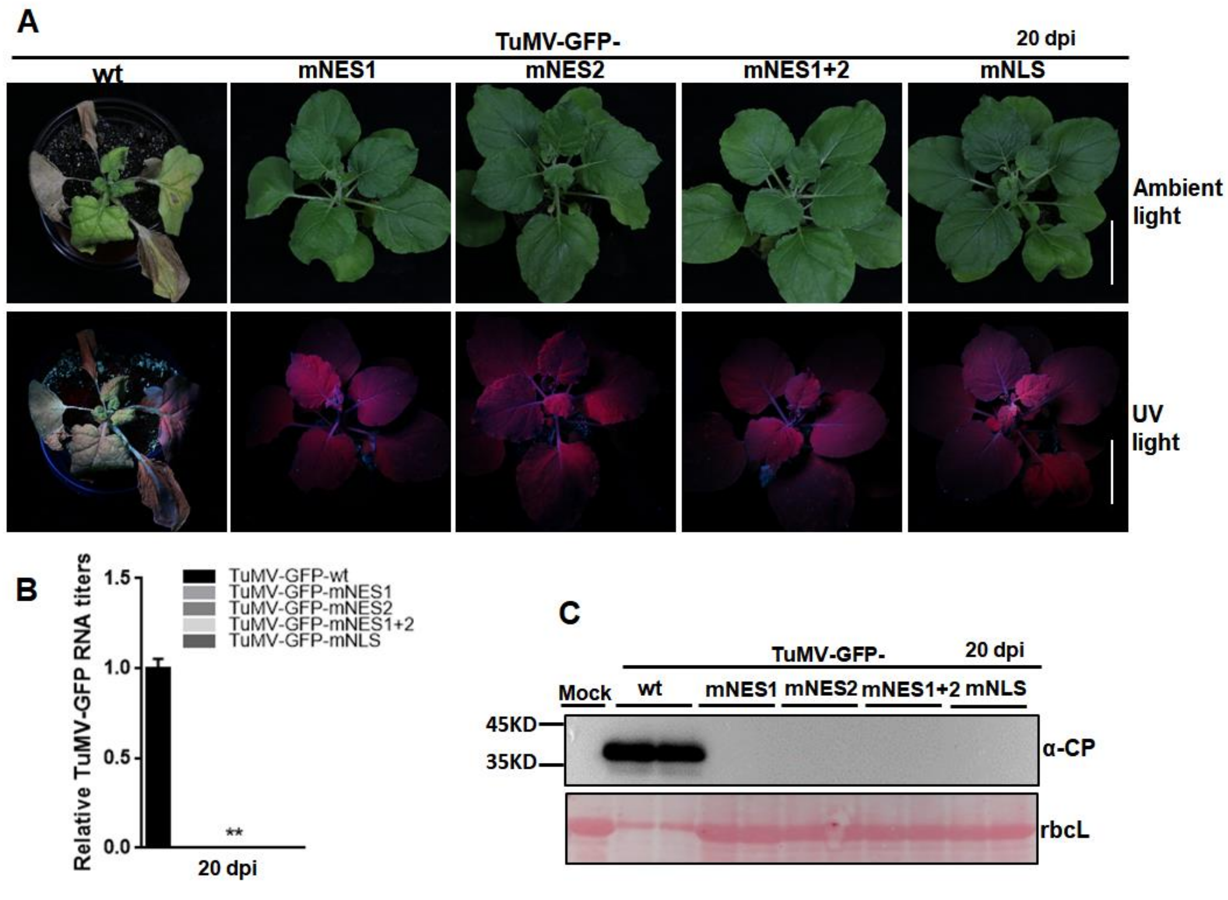

Fig S4. The mutation of functional NESs and NLS of NIb impairs TuMV-GFP systemic infection at 20 dpi. (A) Viral symptoms and GFP fluorescence in plants inoculated with the indicated infectious

clones were photographed under ambient and UV light at 20 dpi. The wild type (wt) TuMV-GFP infection clone, and the TuMV-GFP infection clone harboring the mutation of the functional NES1, NES2, NES1 and NES2 (NES1+2), or NLS were infiltrated onto *N. benthamiana* leaves, and were kept in green house for constant observations. (**B-C**) qRT-PCR and western blot analysis of TuMV-GFP RNA titers and protein accumulation. Total RNA and total protein were extracted from the systemic leaves indicated in (A). Values represent means relative to the leaves infected with wt TuMV-GFP infection clone ± standard deviation (SD) (n =3). The data were analyzed using Student's *t* test and double asterisks indicate $P < 0.01$ *NbActin* was used as an internal control (B). Anti-TuMV coat protein antibodies were used, and the large Rubisco subunit (rbcL) staining with Ponceau S showed the equal loading (C).
